# Field Trapping the Little Fire Ant, *Wasmannia auropunctata*

**DOI:** 10.1673/031.012.9301

**Published:** 2012-08-11

**Authors:** Nathan T. Derstine, Elisa J. Troyer, Caitlyn N. Suttles, Leigh A. Siderhurst, Eric B. Jang, Matthew S. Siderhurst

**Affiliations:** ^1^ Department of Chemistry, Eastern Mennonite University, 1200 Park Rd., Harrisonburg, VA 22802; ^2^ U.S. Pacific Basin Agricultural Research Center, USDA-ARS, P.O. Box 4459, Hilo, HI 96720

**Keywords:** ant detection and monitoring, double—sided tape, microhabitat preferences, one—way trap, pheromone, species—specific trapping, 2,5-dimethyl-3-(2-methylbutyl)pyrazine

## Abstract

Two detection methods for the little fire ant, *Wasmannia auropunctata* (Roger) (Hymenoptera: Formicidae), both employing the pheromone attractant 2,5-dimethyl-3-(2-methylbutyl)pyrazine (2-MeBu-diMePy), were compared with peanut butter based detection, in order to evaluate differences in species specificity and detection reliability. Trapping was conducted using a transect through a macadamia orchard on the island of Hawaii. The transect consisted of a series of three-tree plots, each plot contained a peanut butter coated stick (the most common detection method used for *W. auropunctata* in Hawaii), a one—way trap treated with 2-MeBu-diMePy, and a piece of double-sided tape treated with 2-MeBu-diMePy. While there were no differences in the number of *W. auropunctata* counted with each detection method, and no differences in detection reliability (detecting the known presence of *W. auropunctata* in a plot), the pheromone—incorporating methods showed greater species specificity, retaining *W. auropunctata* almost exclusively. These results demonstrate the potential of pheromone—detection methods to selectively capture target ant species even when other ants are present and abundant. Combined data from all three detection methods and a previous visual survey along the transect showed a marked difference in the frequency of cohabitation among ant species. Of the 10 ant species collected, *W. auropunctata* was found as the sole ant species on a given tree at a significantly higher frequency than all other ant species except *Pheidole fervens*. 94% percent of the trees with *W. auropunctata* had only *W. auropunctata*, supporting previous observations that this species tends to displace other ant species. In addition, *W. auropunctata* microhabitat preferences were investigated using one—way traps containing 2-MeBu-diMePy, which were placed in three arboreal and three non—arboreal locations. While the number of ants captured did not differ by trap placement, when grouped, captures were significantly higher in arboreal than non-arboreal microhabitats.

## Introduction

Considered one of the worst invasive pest ants (Lowe et al. 2000), the little fire ant *Wasmannia auropunctata* (Roger) (Hymenoptera: Formicidae) has negatively impacted both biodiversity and agriculture (Le Breton et al. 2003; Wetterer and Porter 2003; Walker 2006). Its distribution is nearly pantropical, and greenhouse infestations have been reported as far north as Canada and the United Kingdom (Jourdan et al. 2002; Wetterer and Porter 2003). Despite having been present in Florida for approximately 85 years, *W. auropunctata* is not considered a major pest there; on invaded Pacific islands however, it has much larger ecological and economic impacts (Wetterer and Porter 2003).

While *W. auropunctata* workers are rather slow-moving and diminutive in size, the potent venom from their stings is painful, and they have become an important deterrent to farm laborers that harvest infested crops (Smith 1965; Conant 2000). They also affect agriculture by tending homopterans, which directly damage crops and may vector disease (Spencer 1941; de Souza et al. 1998). Ecological impacts include the stressing of vertebrates (Wetterer 1997; Wetterer et al. 1999), reduction of invertebrate populations (Ulloa-Chacón et al. 1991), and displacement of native ants (Le Breton et al. 2003; Walker 2006).

Improved quarantine and prompt eradication of invasive populations are key to controlling the spread of *W. auropunctata* (Wetterer and Porter 2003). Current *W. auropunctata* detection methods employ a food item like peanut butter, which is placed on the ground for an unstandardized length of time and later inspected for the presence of ants (Causton et al. 2005; Kirschenbaum and Grace 2007). However, this technique is not specific to *W. auropunctata* and thus species ID becomes more complicated by the presence of multiple species. For example, peanut butter was used to survey for *W. auropunctata* on the island of Maui (Starr et al. 2007). Although *W. auropunctata* was not detected in the Maui study, over 20 different species of ants were caught, demonstrating the complexity of surveying for a single ant species using a food bait with broad appeal to many ant species. In addition to lacking specificity, food baits are often messy to use, are more susceptible to spoilage, and may be eaten by vertebrates in the field. Given these drawbacks to the use of food baits, there is a need for the development of improved detection methods that increase trapping efficiency and ease—of—use.

Trapping methods are often improved with semiochemical attractants, because these compounds tend to be more species—specific than generic food bait. This is particularly true of pheromones, which usually attract only a single species. Recently, as part of an effort to increase bait specificity, two components of the *W. auropunctata* alarm pheromone were identified as 2,5-dimethyl-3-(2-methylbutyl)pyrazine (2-MeBu-diMePy) and 3-methyl-2-(2-methylbutyl)pyrazine (2-MeBu-MePy) (Showalter et al. 2010). In field and laboratory bioassays, both pyrazines induced attraction and arrestment, and increased locomotion (Troyer et al. 2009; Showalter et al. 2010). The strength and longevity of attractiveness demonstrated by these pheromones is promising for their use in ant detection and control.

Many detection methods improve efficiency and species-specificity by combining the use of an attractant (often a pheromone) with some means of capture (often a trap and/or an adhesive material) (El-Sayed et al. 2006). In a previous study, when 2-MeBu-diMePy was deployed in combination with Tanglefoot, a sticky catch material, groups of *W. auropunctata* were observed to arrest around the adhesive area for up to eight days (Troyer et al. 2009). Ants probed the catch material with their antennae, but demonstrated a strong aversion to venturing onto the coated substrate. These results suggest that Tanglefoot is a poor choice for *W. auropunctata* detection, but do not rule out other adhesive materials as potential capture materials.

Another detection method, which involves combining one—way traps with pheromone, was developed through serendipitous observations of perforated weedmat. While conducting field tests, we observed that ants could squeeze through holes in only one direction. This observation led to the development of a one—way trap prototype, which could provide a new means to capture and retain ants. In addition, the combination of a one-way trap and pheromone may increase species specificity by attracting only *W. auropunctata*. It is expected that other small ants will not be attracted by the pheromone and will have little reason to explore the trap while larger ants will be excluded from the trap by the small hole size in the weedmat. The combination of a one— way trap and an attractant has previously been used to trap tephritid fruit flies in Hawaii, demonstrating the feasibility of this trapping technique (Hiramoto et al. 2006; Jang 2011). However, further testing needs to be conducted to determine the efficacy and specificity of this trapping method for *W. auropunctata* and whether it could be an improvement on current detection methods.

The development of a more efficient and species specific detection method would also allow the investigation of *W. auropunctata* microhabitat preferences. *Wasmannia auropunctata* are known to colonize trees as well as the ground, and are sometimes found in houses (Fernald 1947). Arboreal colonies are often found around the bases of trunks or in crevices, depressions, and crooks formed by branches (Spencer 1941). However, ants have also been known to almost blanket the ground, making nests in any slightly protected area (Spencer 1941; Wetterer and Porter 2003). Undetected arboreal *W. auropunctata* are often difficult to control, since they will recolonize the ground when treatment ends (Vanderwoude and Nadeau 2009). Resolving *W. auropunctata* preferences for arboreal versus non—arboreal microhabitats could help to refine ecological understanding of this ant and aid in optimizing trap placement for detection and control purposes.

This paper presents a series of field experiments comparing *W. auropunctata* detection methods that use either 2-MeBudiMePy as a pheromone attractant or peanut butter as a food lure. Detection methods using the pheromone included either a sticky catch material or a one—way trap. Trap data from these comparisons were also used to evaluate ant species diversity and spatial distribution in the macadamia orchard where the experiments were conducted. Additionally, a vegetation survey of ant microhabitat preferences was conducted, which provided information that would be helpful in the optimization of trap placement for *W. auropunctata* detection/control.

## Materials and Methods

### Insects and field location

**Figure 1.**
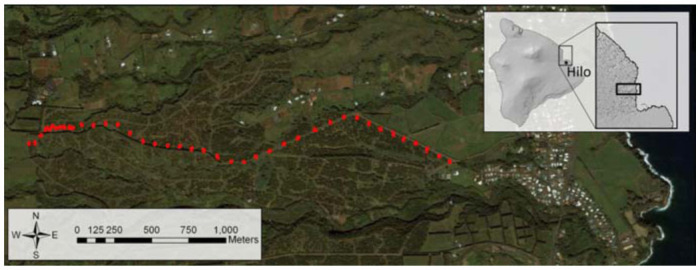
Aerial view of the experimental site, a macadamia orchard outside Papaikou, HI. Red dots mark the transect used in Experiments 2 and 3. High quality figures are available online.

All field tests were conducted in a macadamia orchard on the island of Hawaii, outside Papaikou, HI (GPS coordinates: 19.787029, -155.124443), from 9 June 2010 to 6 July 2010 (Figure 1). Average daily temperatures varied from 20–28 °C and from 71–86% RH. Macadamia trees in this orchard are planted in rows ∼15 m apart. The area directly below the trees is largely free of grass due to herbicide spraying. Rows of trees are spaced at ∼20 m intervals and separated by a border strip of ∼1 m grass.

### Chemicals

A single enantiomer of 2-MeBu-diMePy, 2,5-dimethyl-3-((*S*)-2-methylbutyl)pyrazine (*S*2MeBu-diMePy) was used in Experiment 3. This compound was prepared from (*S*)-1-bromo-2-methylbutane and 3-chloro-2,5-dimethylpyrazine (Sigma-Aldrich, Inc., www.sigmaaldrich.com) using previously described methodology (Showalter et al. 2010). Experiment 4 used the racemic *W. auropunctata* alarm pheromone component 2-MeBu-diMePy, which was prepared as described by Showalter et al. (2010). All solvents and reagent compounds were purchased from Sigma-Aldrich, Inc. or Fisher Scientific (www.fishersci.com). Lures used in Experiments 3 and 4 were made by loading 1 mg of pheromone in 100 µL CH2Cl2 onto rubber septa with a glass syringe. Controls were loaded with 100 µL CH_2_Cl2 only.

### Detection methods

Two new detection methods were developed for comparison with the existing food—based detection of *W. auropunctata*. Both methods included a pheromone attractant and a capture/retention device; either a sticky trap or a one—way trap. The sticky trap utilized double—sided carpet tape, and the one—way trap was constructed from plastic Petri dishes and weed mat.

**Figure 2.**
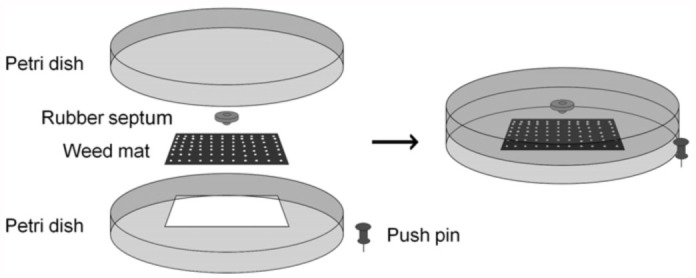
Assembly diagram for one-way traps. Pushpins, septas, and weedmat pieces were attached with hotmelt glue. The Petri dish pieces were fitted together without adhesive. High quality figures are available online.

One—way traps were assembled as follows (Figure 2). The bottom part of the trap was constructed from a Petri dish bottom (100 O.D. × 15 mm H) (BD Biosciences, www.bdbiosciences.com) with a 5 × 5 cm square cut from the middle. Conically—perforated weed mat (Weed Block, Easy Gardener Products, Inc., www.easygardener.com) was affixed over the opening with hot—glue. A push pin was glued onto the outside of the Petri dish to secure the trap to trees at test sites. The top part of the trap consisted of a second Petri dish bottom, with a rubber septum (13 mm snap—on stopper rubber septa, Wheaton, www.wheaton.com) glued onto the inside surface, which fit snugly onto the bottom half of the trap. The rubber septa were treated with pheromone in the field, and trap halves were fitted together immediately before each experiment.

The sticky trap was made from a 3.5 × 3.5 cm square of double—sided outdoor carpet tape (3M, www.3m.com) with a rubber septum stuck to the center of the outward—facing surface. The tape was precut and rubber septa were applied in the laboratory. The rubber septa were treated with pheromone in the field immediately before each experiment.

The food—based detection method involved the use of a popsicle stick thinly coated with peanut butter attached to trees with a push pin. The peanut butter was applied in the field immediately before each experiment.

### Experiment 1

Experiment 1 assessed different sticky catch materials for possible use in combination with a pheromone attractant. Specifically, 10 different types of tape were compared for their effectiveness in capturing *W. auropunctata*. Tests were performed in trees with easily observed *W. auropunctata* trails. Each tape was tightly wrapped around a branch with the sticky side exposed. Only one piece of tape was affixed to a single branch. After 24 hours, an assessment (by direct count or estimation) of the number of ants stuck to each piece of tape was recorded. Evaluated tapes were: duct tape (3M), electrical tape (3M), blue painters tape (3M), outdoor carpet tape (3M), clear string reinforced tape (3M), label tape (Thermo Fisher Scientific, www.thermofisher.com), scotch tape (3M), brown packing tape (3M), masking tape (3M), double—sided poster tape (3M), and double—sided scotch tape (3M).

### Experiment 2

Experiment 2 was an ant survey based on visual observation and collection. The purpose of this survey was to provide baseline information on ant species diversity and distribution at the study site, while creating the transect used in later experiments. Initially, an aerial photograph was used to designate a transect line for sampling from one end of the orchard to the other. 41 plots of three trees were then selected and surveyed along the transect (Figure 1). Trees within a plot were selected based on similarities in apparent ant diversity and microclimate. Ants from surveyed trees were collected and placed in labeled vials for later identification. To increase the number of plots containing *W. auropunctata* within the transect, additional plots were selected every third row in areas with observed *W. auropunctata* populations. In areas without *W. auropunctata*, plots were chosen from every 10th row. This increased the number of plots with *W. auropunctata* to roughly one—third of the total test plots. During the survey, an isolated population of *W. auropunctata* was found (plot 30). In this instance, the three trees selected were positive for *W. auropunctata*, and adjacent plots were 10 rows apart. GPS coordinates were recorded for all selected trees in each plot. Ants were identified to species in the laboratory based on adult morphology using several keys (Huddleston et al. 1968; Fernández and Guerrero 2008; Discovery Life 2011; Krushelnycky 2011). To confirm identifications, voucher specimens were sent to the Plant Pest Control Branch of the Hawaii Department of Agriculture.

### Experiment 3

Experiment 3 compared the species specificity and detection reliability of the three potential *W. auropunctata* detection methods. The three detection methods were a one-way trap with a pheromone attractant (*S*2-MeBu-diMePy), double—sided tape with a pheromone attractant, and a peanut butter—coated popsicle stick. These detection devices were deployed, one per tree, within the plots previously selected in Experiment 2. The order in which the detection devices were deployed was rotated within each sequential plot. Detection methods were deployed in the early morning and were collected in plastic bags after 24 hours. After retrieval, traps were frozen and ants were identified and counted.

### Experiment 4

Based on the success of the one—way trap detection method used in Experiment 3, Experiment 4 aimed to investigate *W. auropunctata* microhabitat preferences in order to determine optimal one-way trap placement. Replicate plots were selected in areas containing *W. auropunctata*. Each replicate plot contained arboreal placements in macadamia trees, which were subdivided into branch, crook (branch/trunk junction), and the foot of the tree, and non—arboreal, which were subdivided into ground (under tree canopy), grass (tall grass between tree rows), and scrub (overgrown areas adjacent to the orchard). Traps were deployed in six replicate plots for 24 hours before being collected in plastic bags and frozen. Ants were then counted in the laboratory.

### Statistical analysis

Data from transect visual survey (Experiment 2) and trapping comparison (Experiment 3) were mapped using GIS software. For detection method comparisons, species specificity is defined as detecting the known presence of *W. auropunctata* (based on data from Experiment 2) without capturing other species. Differences in species specificity between detection methods were analyzed using Fisher's exact test. Ant species diversity (cohabitation) within plots was analyzed using Fisher's exact test, since many of the species had small sample sizes. Initially, *W. auropunctata* cohabitation was compared to all other ant species by collapsing the ant species categories into a 2 × 2 contingency table. Cohabitation for all ant species were then compared in 2 × 2 contingency tables with Fisher's exact test. Multiple hypothesis testing error was measured by false discovery rate (FDR) using the False Discovery Rate Tool (Microsoft, www.microsoft.com). The number of *W. auropunctata* captures with each detection method (Experiment 3) were analyzed using ANOVA followed by Tukey's HSD test (α = 0.05) to compare means. The number of other ant captures with each detection method (Experiment 3) were not homogeneous (Levene's test) and were analyzed by Kruskal—Wallis test followed by pair—wise comparisons with Mann—Whitney U test to compare medians. The number of *W. auropunctata* trapped in different microhabitats (Experiment 4) was not homogeneous (Levene's test) and was analyzed by Kruskal—Wallis test. Additionally, for the vegetation survey, arboreal and non—arboreal placements were grouped and compared with a Mann—Whitney U test. All analyses of significance were made at the *p* < 0.05 level. Statistical analyses other than FDR were performed using SPSS version 15.0 (SPSS, Inc., www.ibm.com).

## Results

Experiment 1 compared 10 types of tape for capturing and retaining *W. auropunctata*. Tape types that retained ants (2–500 ants per piece of tape) were clear string reinforced, outdoor carpet, colored label, duct, masking, and electrical (ranked highest to lowest). Of these, the double—sided outdoor carpet tape was chosen for further testing based on the comparatively high number of ants captured and the ease of field placement for testing. Tapes that did not retain ants were scotch tape, blue painter's tape, double—sided poster tape, double—sided scotch tape, and brown packing tape. In several instances, ants were observed to walk unimpeded across this latter group of tapes.

**Figure 3.**
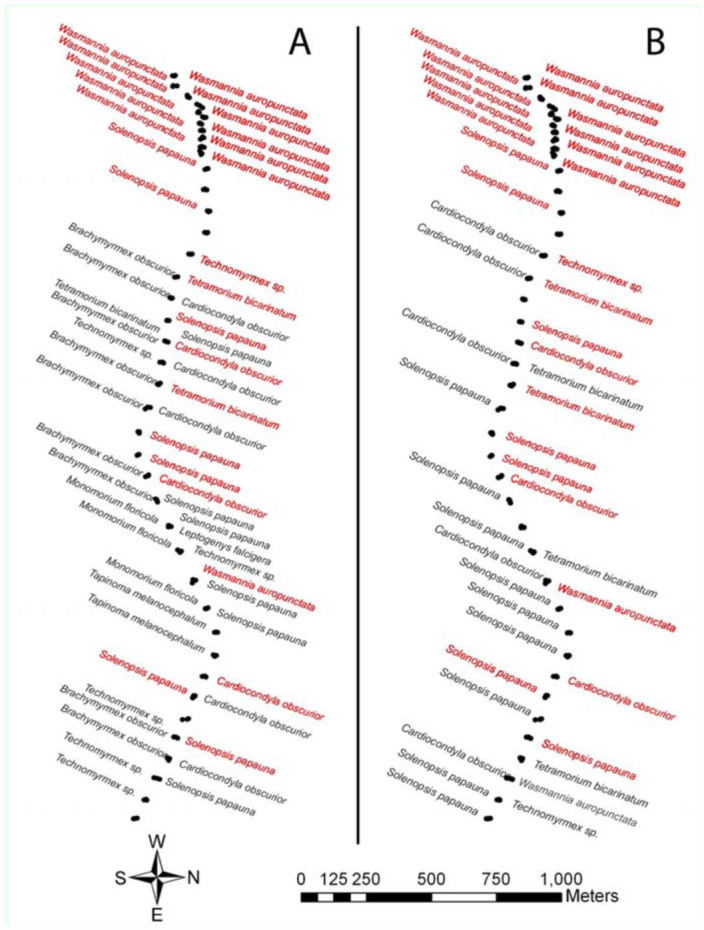
Ant species collected in transect plots. (A) Experiment 2, visual survey/collection. (B) Experiment 2, combined ant captures (peanut butter, one—way trap, and double—sided tape). Red labels indicate that the same ant species was collected (Exp. 2) and trapped (Exp. 3) on the same tree during both experiments. High quality figures are available online.

**Figure 4.**
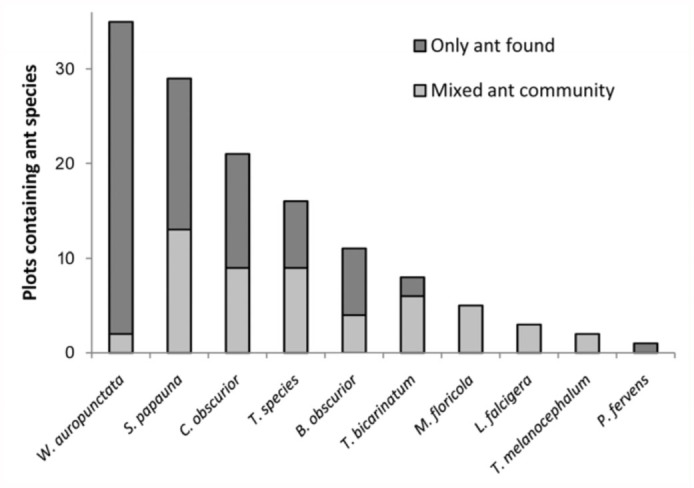
Ant species diversity (cohabitation) data. *Wasmannia auropunctata* was found as the lone ant species in a plot at a significantly higher frequency (*p* > < 0.05, Q < 0.001) than all other ant species except *Pheidole fervens* (Fisher's exact test with FDR analysis). High quality figures are available online.

Experiment 2 assessed the diversity of ant species along a transect by means of a visual survey (Figures 1 and 3). From tree collections, 10 ant species were identified. These were *Brachymyrmex obscurior* Forel, *Cardiocondyla obscurior* Wheeler, *Leptogenys falcigera* Roger, *Monomorium floricola* (Jerdon), *Pheidole fervens* Smith, *Solenopsis papuana* Emery, *Tapinoma melanocephalum* (Mayr), *Technomyrmex* sp. (v*itiensis* (Emery) and/or *albipes* Mann), *Tetramorium bicarinatum* (Mayr), and *W. auropunctata* (Roger). With the exception of plots 30 and 39, *W. auropunctata* was found exclusively in the western end of the transect (Figure 3). Additionally, when *W.* *auropunctata* was found on a tree, it was the only species collected from that tree 94% of the time. *Wasmannia auropunctata* was found as the lone ant species at a significantly higher frequency than all other ant species except *P. fervens* (*p* < 0.05, *Q* < 0.001) (Figure 4).

**Figure 5.**
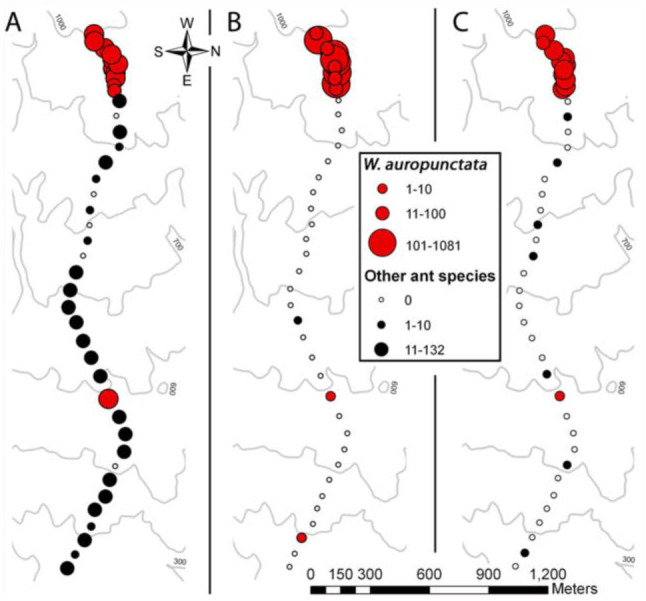
Experiment 3, number of ants caught by the three detection methods: (A) Peanut butter, (B) one–way traps, (C) double—sided tape. Red circles indicate plots where *Wasmannia auropunctata* were detected, black circles indicate plots where other ant species were detected. High quality figures are available online.

**Figure 6.**
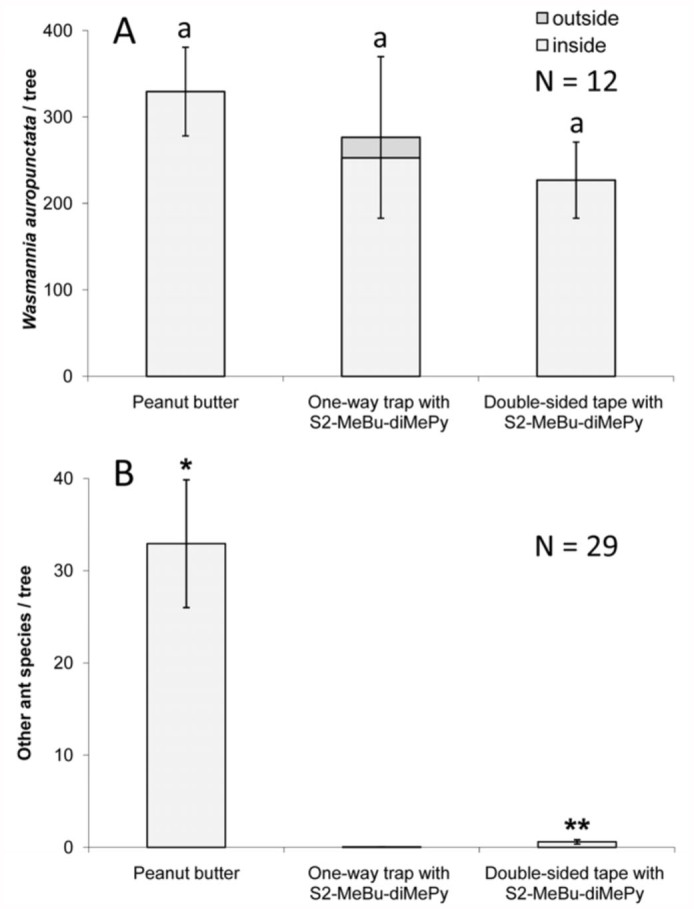
Experiment 3, numbers (mean ± SE) of ants captured per tree. (A) Traps capturing *Wasmannia auropunctata*, primarily at the west end of the transect. Letters represent significant differences (*p* < 0.05) between arrestments and crossings of different treatments (ANOVA, followed by Tukey's HSD). (B) Traps capturing other ant species, primarily in the middle and east end of the transect. * Median significantly different than other medians (Kruskal—Wallis, followed by Mann—Whitney U test). ** Median significantly different than one—way trap (Kruskal—Wallis, followed by Mann—Whitney U test). High quality figures are available online.

Experiment 3 compared three *W. auropunctata* detection methods (Figures 5 and 6). In plots where *W. auropunctata* was detected during the visual survey (Experiment 2), the number of ants caught per tree did not vary significantly between detection methods (ANOVA, *p* = 0.558) (Figure 6A). In plots where *W. auropunctata* was not detected during the visual survey, the one—way trap and double—sided tape, which both used a pheromone attractant, caught significantly fewer ants of all species than the peanut butter detection method (pair—wise Mann—Whitney U test, *p* < 0.01) (Figure 6B). In one instance (plot 39), the one—way trap detected previously unobserved *W. auropunctata* (Figure 5). There was one instance (plot 26) where *C. obscurior* was found inside the one—way trap, seven instances of species other than *W. auropunctata* caught with the doublesided tape, and 25 instances of species other than *W. auropunctata* on the peanut butter coated stick. Eight of the 41 peanut butter coated sticks (originally placed in trees) were recovered from the ground, some of which no longer had peanut butter. Presumably they were knocked down by vertebrate wildlife (i.e., mongooses, pigs, birds) and licked clean. The one—way trap and double—sided tape captures were 98% and 83% species specific (detecting the known presence of *W. auropunctata* without capturing other species), respectively. While there were no significant differences between the species—specificity of captures with the one—way trap or double—sided tape captures (*p* = 0.0571), they were both significantly more selective than peanut butter (39%) (*p* < 0.01 for both).

**Figure 7.**
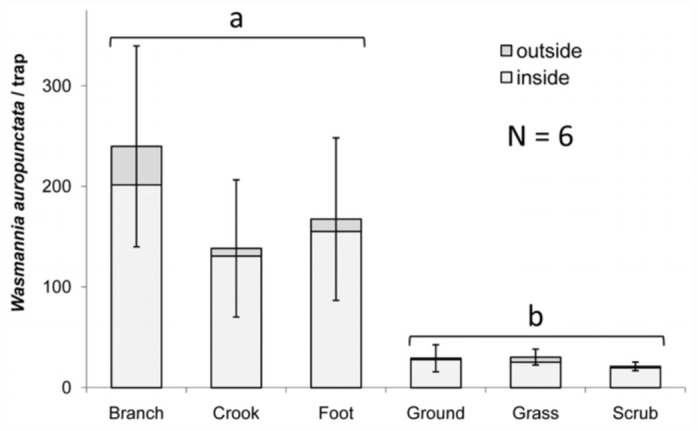
Experiment 4, numbers (mean ± SE) of *Wasmannia auropunctata* captured in one—way traps in different microhabitats. Letters represent significant differences (*p* < 0.05) between arboreal and non—arboreal placement groupings (Kruskal—Wallis, followed by Mann—Whitney U test). High quality figures are available online.

Experiment 4 investigated *W. auropunctata* microhabitat preferences with one-way trap placement (Figure 7). *Wasmannia auropunctata* were detected in all microhabitats. When analyzed individually, the mean number of *W. auropunctata* caught differed significantly among microhabitats (Kruskal—Wallis test, *p* < 0.05). When trap placements were grouped as arboreal or non—arboreal, the tree-associated traps caught significantly more *W. auropunctata* than those located further from the tree (Mann—Whitney U test, *p* = 0.01).

## Discussion

The assessment of different sticky catch materials for use in *W. auropunctata* detection (Experiment 1) suggested that tested tapes fall into two groups; materials which *W. auropunctata* will traverse and sometimes become stuck to, and those which they will traverse without becoming stuck. A third group of materials are those that deter *W. auropunctata* from traversing, presumably because they are highly adhesive and are perceived as such by the ants. An example of this third group of sticky materials is Tanglefoot, which acts as a barrier that *W. auropunctata* will not cross or become entangled in when 2-MeBu-diMePy is present as an attractant (Troyer et al. 2009). These observations of *W. auropunctata* behavior and locomotion with regard to sticky materials suggest that adhesives should be of intermediate strength so as not to deter ants from trying to cross while being sticky enough to entangle ants during crossing.

Survey data (Experiment 2) supports previous research describing the ability of *W.* *auropunctata* to displace other ant species (Le Breton et al. 2002; Armbrect and Ulloa-Chacon 2003; Le Breton et al. 2003; Kirschenbaum and Grace 2007). Of all ants surveyed, the data show *W. auropunctata* to be the least likely to co—inhabit with another ant species in a given tree (Figure 4). The majority of the other ant species were consistently found with 2-3 other species on the same tree.

Field studies of *W. auropunctata* detection methods (Experiment 3) showed increased species specificity when the pheromone 2-MeBu-diMePy was used in combination with a one—way trap or double—sided tape (Figure 5 and 6). While all methods succeeded in detecting *W. auropunctata*, only the methods using 2-MeBu-diMePy were significantly more species—specific; detecting the known presence of *W. auropunctata* without capturing other species. Multiple ant species were observed feeding on peanut butter—coated sticks, and interference from larger animals (presumably mongoose, pigs, or birds) made field testing problematic. Similar problems were encountered in *W. auropunctata* surveying attempts using peanut butter in the Galapagos (Abedrabbo 1994). Additionally, comparisons between visual survey and trapping data confirmed the detection reliability of one—way traps and double—sided tape used in combination with 2-MeBu-diMePy; *W. auropunctata* was caught wherever it had been previously observed, with one additional occurrence detected with the one—way trap (Figure 3). These results demonstrate the potential of pheromone detection methods to selectively capture target ant species even when other ants are present and abundant.

Of particular interest to regulatory agencies, pheromone aided detection may decrease the need for taxonomic identification of field samples if only target ant species are attracted to the detection device. There are two commonly used ant monitoring methods: a broad survey of taxa diversity and the targeted detection of single species (often invasive pests) to aid in control. Surveys for ant taxa diversity commonly rely on pitfall traps, baited traps, and leaf—litter extractions (or some combination thereof) for collections (Botes et al. 2006; Neville et al. 2008; Ouellette et al. 2010). This requires trained taxonomists to sort through large sample quantities of entire invertebrate assemblages. Thus, the aforementioned survey methods are utilized with great inefficiency towards the purpose of monitoring a single target species, be it for pest control, tracking the spread of invasive species, or emerging applications like biodiversity monitoring. For the latter, Andersen (2004) describes the dearth of species—level tools for non—specialists as a primary hindrance to implementing biodiversity monitoring programs. However, the high species specificity of both the one— way trap and the sticky tape, in combination with an attractive pheromone, has the potential to be such a tool. Pushing non—specialist detection to an extreme, species—specific trapping might even be applicable to citizen/scientist collaboration monitoring specific ant species (Braschler 2009). Using pheromone traps would enable members of the public to simply report the presence or absence of ants without creating a large number of samples to be examined.

While the one—way trap and the double—sided tape performed similarly in the field, there are differences between the two methods that should be considered in assessing which is better suited for *W. auropunctata* monitoring. From a design standpoint, the simplicity of the double—sided tape is appealing. However, over longer test periods the sticky surface is likely to become covered with detritus from the environment, which would decrease the capacity to retain attracted *W. auropunctata*. The one—way trap has the advantage of excluding insects ∼1 mm and much of the environmental detritus, which might affect the tape. Additionally, even though species specificity was not significantly different between these two detection methods, it is our opinion that the one—way trap has the potential to be more selective long—term. The one—way trap also has the potential to capture many more ants given the larger volume inside the trap. Beyond use with *W. auropunctata*, this trap has the potential to be used with other ant pheromones, providing a model for species specific monitoring of small (1–2 mm) ants.

Results from the assessment of several microhabitats within the macadamia orchard (Experiment 4) suggested that the optimal location for one—way trap placement is in trees. Although all traps caught *W. auropunctata*, one—way traps in trees or near the foot of macadamia trees caught more ants than those on the ground or in grass or shrub thickets. This speaks to the near ubiquity of *W. auropunctata* in infested areas, and the preference of *W. auropunctata* populations for tree-based microhabitats in the macadamia orchard.

## Abbreviations

**2-MeBu-diMeP**,: pheromone attractant 2,5-dimethyl-3-(2-methylbutyl)pyrazine;
**2-MeBu-MePy**,: pheromone attractant 3-methyl-2-(2-methylbutyl)pyrazine;
**S2-MeBu-diMePy**,: 2,5-dimethyl-3-((S)-2- methylbutyl)pyrazine, an enantiomer of 2-MeBu-diMePy
